# DeepCellEss: cell line-specific essential protein prediction with attention-based interpretable deep learning

**DOI:** 10.1093/bioinformatics/btac779

**Published:** 2022-12-02

**Authors:** Yiming Li, Min Zeng, Fuhao Zhang, Fang-Xiang Wu, Min Li

**Affiliations:** Hunan Provincial Key Lab on Bioinformatics, School of Computer Science and Engineering, Central South University, Changsha 410083, China; Hunan Provincial Key Lab on Bioinformatics, School of Computer Science and Engineering, Central South University, Changsha 410083, China; Hunan Provincial Key Lab on Bioinformatics, School of Computer Science and Engineering, Central South University, Changsha 410083, China; Division of Biomedical Engineering, Department of Computer Science, Department of Mechanical Engineering University of Saskatchewan, Saskatoon, SK S7N 5A9, Canada; Hunan Provincial Key Lab on Bioinformatics, School of Computer Science and Engineering, Central South University, Changsha 410083, China

## Abstract

**Motivation:**

Protein essentiality is usually accepted to be a conditional trait and strongly affected by cellular environments. However, existing computational methods often do not take such characteristics into account, preferring to incorporate all available data and train a general model for all cell lines. In addition, the lack of model interpretability limits further exploration and analysis of essential protein predictions.

**Results:**

In this study, we proposed DeepCellEss, a sequence-based interpretable deep learning framework for cell line-specific essential protein predictions. DeepCellEss utilizes a convolutional neural network and bidirectional long short-term memory to learn short- and long-range latent information from protein sequences. Further, a multi-head self-attention mechanism is used to provide residue-level model interpretability. For model construction, we collected extremely large-scale benchmark datasets across 323 cell lines. Extensive computational experiments demonstrate that DeepCellEss yields effective prediction performance for different cell lines and outperforms existing sequence-based methods as well as network-based centrality measures. Finally, we conducted some case studies to illustrate the necessity of considering specific cell lines and the superiority of DeepCellEss. We believe that DeepCellEss can serve as a useful tool for predicting essential proteins across different cell lines.

**Availability and implementation:**

The DeepCellEss web server is available at http://csuligroup.com:8000/DeepCellEss. The source code and data underlying this study can be obtained from https://github.com/CSUBioGroup/DeepCellEss.

**Supplementary information:**

Supplementary data are available at *Bioinformatics* online.

## 1 Introduction

Essential genes are indispensable for the survival of a single-celled organism, a cell line or a multicellular organism (Bartha *et al.*, 2018). Essential proteins are products of essential genes, which perform the basic functions in the biological processes, and can be used to facilitate drug discovery and disease treatment (Ji *et al.*, 2019). The traditional biological experiments of essential protein identification include transposon mutagenesis, single-gene knockout, RNA interference and recent CRISPR gene-editing technology (Peters *et al.*, 2016; Rancati *et al.*, 2018). However, these wet-lab experiments are expensive, time-consuming and labor-intensive. Thus, it is urgent to develop effective and accurate computational methods to predict essential proteins.

The computational methods can be roughly divided into two categories: network-based centrality measures and machine learning-based methods. Network-based centrality measures usually rely on a constructed biological network and design a scoring function to assign essential scores for each node in the constructed biological network. The Centrality-Lethality Rule was first proposed by Jeong *et al.* (2001), which points out highly connected proteins in a protein–protein network are more likely to be essential proteins. After that, a lot of network-based centrality measures such as betweenness centrality (BC), closeness centrality (CC), eigenvector centrality (EC), local average centrality (LAC) and maximum neighborhood component (MNC) were proposed to identify essential proteins (Li *et al.*, 2016; Lin *et al.*, 2008). Considering that some biological information is very important for protein essentiality, researchers incorporated various biological information sources in their scoring functions, including protein subcellular localization information, gene expression profiles, orthologous information and RNA-Seq data (Lei *et al.*, 2018; Li *et al.*, 2014, 2016; Tang *et al.*, 2014).

With the rapid development of high-throughput sequencing technology, more and more essential protein data are accumulated, which provide a data foundation of machine learning-based methods. Deng *et al.* (2011) proposed a machine learning-based integrative model that uses Naïve Bayes, logistical regression, C4.5 decision tree and CN2 rule to estimate essentiality. Guo *et al.* (2017) adopted a support vector machine (SVM) to construct a prediction model from nucleotide composition and association information. Kuang *et al.* (2021) developed a machine learning model which combines gradient-boosted tree, SVM and multi-layer perceptron (MLP) to predict essential genes. Zeng *et al.* (2021) developed an ensemble deep learning model by integrating multiple gradient boosting decision tree (GBDT) base classifiers for accurate prediction. Recently, deep learning techniques have achieved great success in the bioinformatics field (Eraslan *et al.*, 2019). Inspired by their success, some researchers designed deep-learning models to predict essential proteins. For instance, Zeng *et al.* (2019) applied deep learning techniques to predict essential proteins by integrating protein–protein interaction (PPI) networks, gene expression profiles and subcellular localization data. Hasan and Lonardi (2020) utilized a MLP to develop a deep learning model for essentiality prediction from sequence-derived features. Zhang *et al.* (2020) proposed DeepHE, a deep learning model to predict human essential genes by integrating features derived from PPI networks and sequences. Li *et al.* (2021) developed an ensemble deep learning model, EP-EDL, which applied convolutional neural networks (CNN) to predict human essential proteins from evolutionary information.

Although a lot of computational methods have been proposed, they still suffer from some limitations. First, accumulated evidence reveals that the protein essentialities are highly related to cellular environments, which means proteins show different essentiality in different cell lines (Behan *et al.*, 2019). Most of the existing computational methods do not take cell line-specificity into account. They often merge essential protein data from multiple cell lines with different labels into a single unified dataset to conduct model training, which fails to accurately identify essential proteins in diverse cell lines. Second, most of the existing machine/deep learning-based methods only focus on improving the prediction performance but fail to give an interpretation for their prediction results. The lack of interpretability makes their models become black boxes, which limits the understanding of their models for biologists. Therefore, developing an interpretable model is very important for the practical applications of computational methods.

To address the above limitations, we proposed DeepCellEss, a cell line-specific deep learning-based essential protein predictor with the attention mechanism. To create a cell line-specific model, we collected extremely large-scale datasets including 16 408 proteins across 323 different cell lines to train and test DeepCellEss. DeepCellEss uses CNN to extract local features from protein sequences, and then applies the multi-head self-attention mechanism to enhance weights from CNN and provide model interpretation. Then, these enhanced signals are fed into a bidirectional long short-term memory (bi-LSTM) to capture long-range dependencies between residues. Finally, a fully connected layer with a sigmoid function performs the classification task.

We conducted extensive experiments to evaluate the performance of DeepCellEss. In comparison, DeepCellEss shows greater effectiveness in predicting essential proteins than existing sequence-based methods. Compared to network-based centrality measures under cell line-specific networks, the results demonstrate that DeepCellEss effectively compensates for the limitations of network-based centrality measures. Furthermore, we performed some case studies which show the advantages of taking cell line-specificity into consideration. In addition, we carried out ablation studies to demonstrate the benefits of our proposed network architecture. Finally, we built a user-friendly webserver to expand our tool's accessibility.

## 2 Materials and methods

### 2.1 Data collection

**Fig. 1.**
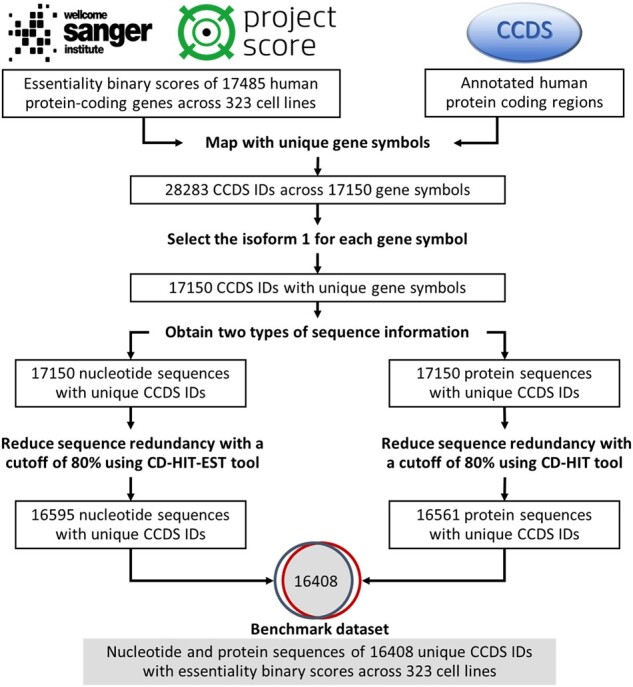
Data collection process of large-scale cell line-specific protein essentiality datasets

To construct a practical cell line-specific prediction model, we collected protein essentiality data in extremely large-scale cell lines. Figure 1 shows the collection process of our cell line-specific benchmark datasets, which can be described as follows:

1. We downloaded the essentiality data generated by the Wellcome Sanger Institutes (Release 1) from the Project Score database (Dwane *et al.*, 2021). The data were identified from a large number of systematic genome-scale CRISPR-Cas9 drop-out screens, including varying binary essential scores for 17 485 human protein-coding genes in 323 different human cell lines. The score of 1 refers to essential and 0 refers to non-essential.
2. We collected sequence information from the Consensus CoDing Sequence (CCDS) database (Release 22) (Pruitt *et al.*, 2009) by mapping with unique gene symbols. In previous sequence-based methods, nucleotide-level and protein-level sequences have been used in essentiality prediction task, thus we collected both of them for further comparison and analysis. If one gene could match more than one protein sequence, we chose the sequence of the first annotated protein isoform produced by this gene as its corresponding protein sequence.
3. We used CD-HIT and CD-HIT-EST (Li and Godzik, 2006) to remove the redundant sequences at the protein-level and nucleotide-level datasets, respectively. The sequence identity cutoff is set to 0.8, which means the remaining samples have sequence similarity less than 80% in both nucleotide-level and protein-level sequences.

Based on the above processes, the resulting benchmark dataset comprises the binary essentiality labels and the sequence information of 16 408 proteins across 323 cell lines, which is the foundation of our sequence-based cell line-specific prediction models.

### 2.2 Cell line-specific essentiality data analysis

**Fig. 2.**
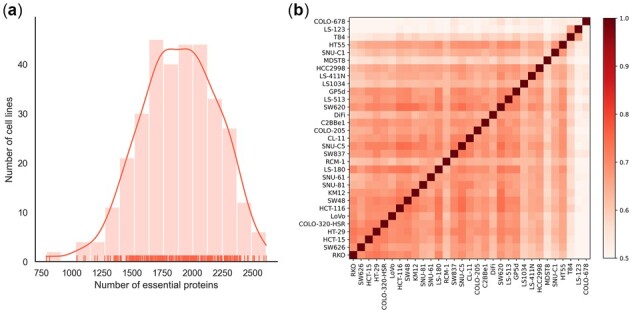
Analysis of protein essentiality across different cell lines. (**a**) Distribution of the numbers of essential proteins across 323 cell lines. (**b**) Heatmap of pairwise Pearson correlation coefficients for essential protein data across 31 different colorectal carcinoma cell lines. Darker color means the two cell lines have more common essential proteins

We performed some analysis to illustrate the significant differences of essential proteins across cell lines. Figure 2a shows the distribution of essential protein numbers of 323 cell lines, which indicates a wide span in the essential protein number across different cell lines, with the COLO-678 cell line in the large intestine tissue having the fewest essential proteins (742) and the A2780ADR cell line in the lung tissue having the most (2491). The average number of is 1799, accounting for 10.96% of the total 16 408 proteins. In addition, to investigate the correlation between essential proteins in different cell lines, we took colorectal carcinoma as an example for analysis. In our collected dataset, colorectal carcinoma has 31 different cell lines. We used Pearson correlation coefficients (PCCs) to represent the similarities of essential protein list for a pair of cell lines. The heatmap in Figure 2b shows the correlation between essential proteins across 31 colorectal carcinoma cell lines (the heatmaps of other cancer types with more than 10 cell lines are shown in Supplementary Fig. S1), where darker color means the two cell lines have more common essential proteins. From it, we can see that although these 31 cell lines belong to the same cancer (colorectal carcinoma), the essential proteins of them are quite different. The differences in essential proteins in different cell lines shown in Figure 2 drove our work on the construction of cell line-specific models.

### 2.3 Model architecture

**Fig. 3.**
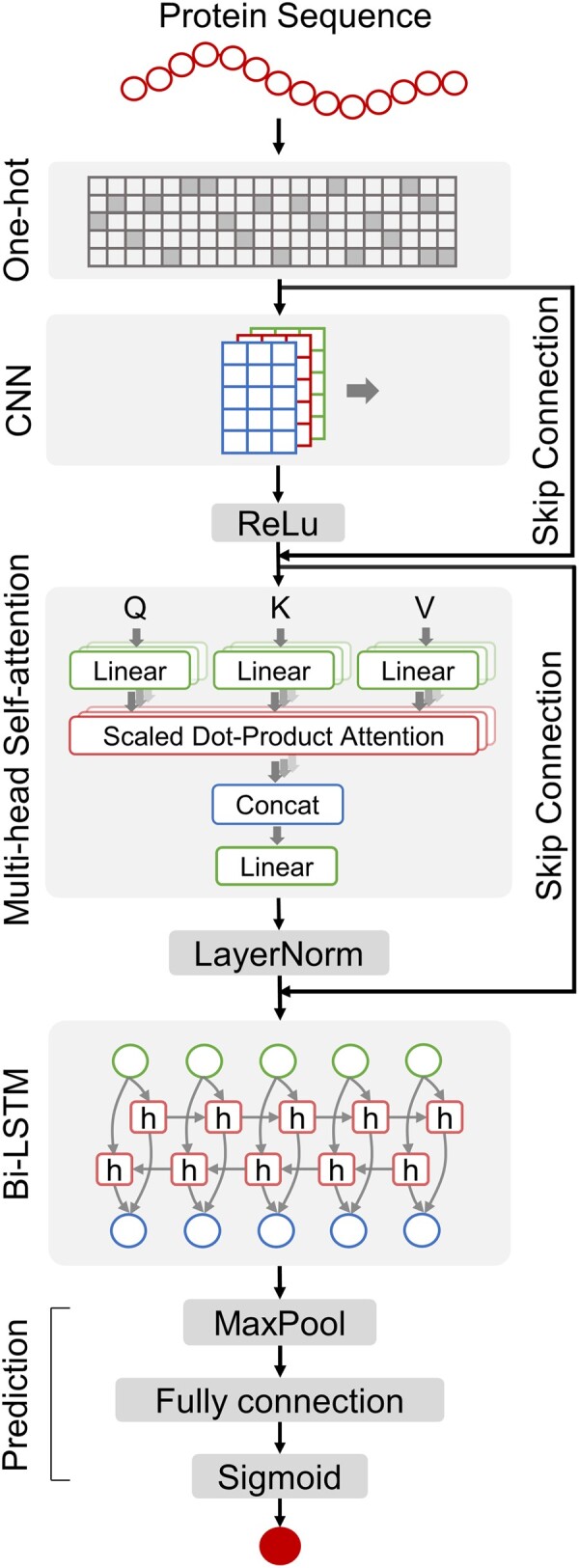
DeepCellEss framework. DeepCellEss accepts a protein sequence as input and converts it into a numerical matrix using one-hot encoding. After that, a CNN module is employed to effectively capture sequence local information. The multi-head self-attention is used to produce residue-level attention scores for model interpretability. Additionally, two skip-connection operations are implemented around CNN and the multi-head self-attention to avoid the model degradation problem. After multi-head self-attention, a bi-LSTM module is applied to model sequential data by learning long-range dependencies. Finally, the prediction task is performed after a max-pooling and fully connected layer

DeepCellEss is a sequence-based end-to-end deep learning prediction model. The overview of DeepCellEss is presented in Figure 3, which consists of five modules i.e. sequence representation, CNN, multi-head self-attention, bi-LSTM and prediction. The detailed descriptions of the five modules are as follows.

#### 2.3.1 Sequence representation

The sequence representation module converts the raw protein sequences of variant lengths into fixed-size numeric feature matrices through one-hot encoding method. Formally, given a protein sequence $S=\left\{a_{1},a_{2},a_{3},\ldots ,a_{L}\right\}$, where *L* means the length of the sequence, $a_{i}$ represents the residue at position i. There are 21 possible $a_{i}$ in a protein sequence i.e. 20 types of standard protein residues and others. By using one-hot encoding, each type of residue is encoded into a 21D binary vector $\vec{x}$. Hence, each protein sequence can be represented numerically as $X=\left\{{\vec{x}}_{1},{\vec{x}}_{2},\ldots ,{\vec{x}}_{L}\right\}$ and fed into the next model module.

#### 2.3.2 Convolutional neural networks

We applied a CNN module to extract latent local knowledge from the raw protein sequences. CNN is a very popular class of neural networks in the fields of computer vision and natural language processing, and have been successfully applied to many bioinformatics prediction problems (Kim, 2014; Zeng *et al.*, 2020). Because of parameter sharing and local connectivity, CNN is able to learn dependencies between adjacent residues effectively. Numerous convolution kernels slide along the sequential features and capture important patterns thus delivering features enriched with local knowledge. In DeepCellEss, we employed a 1D-convolution layer after sequence representation and then followed by a rectified linear unit (ReLU) activation function. Thus, we can obtain an output representation with local information.

#### 2.3.3 Multi-head self-attention

After CNN, a multi-head self-attention module is utilized in DeepCellEss (Vaswani *et al.*, 2017). This module has two primary functions. On the one hand, it can enhance the functionality of CNN module by compensating for its limitation of locality. Instead of using a pure CNN module, the combination of CNN and self-attention helps the model to focus on important sequence regions within a larger scope. Specifically, the output of a single-head self-attention is computed as

(1) $$\begin{array}{c} SA\left(Q,\;K,V\right)=\mathrm{softmax}\left(\frac{\;QK^{T}}{\sqrt{d_{k}}}\right)V \end{array}$$

where Q, K and V represent the query, key and value, respectively. $\frac{1}{\sqrt{d_{k}}}$ are the scaling factor of the dot-product attention. The $\mathrm{softmax}\left(\cdot \right)$ stands for the softmax operation.

Additionally, because it is hard to learn the representation from various perspectives by using self-attention with a single head, we introduced the self-attention with multiple heads to capture more informative features. Thus, the output of the multi-head self-attention module is

(2) $$\begin{array}{c} MA=\mathrm{norm}\left(\mathrm{concat}\left({SA}_{1},{SA}_{2},\ldots ,{SA}_{h}\right)W^{O}\right) \end{array}$$

where h is the number of heads, $W^{O}$ is a learnable parameter matrix, concat$\left(\cdot \right)$ stands for the concatenation operation, norm$\left(\cdot \right)$ stands for the layer normalization operation to maintain the stability of data distribution and better model training.

On the other hand, the self-attention mechanism enables our model to explain prediction results from interpretable attention score distributions. The details of model interpretability can be found in Section 2.4. Through the multi-head self-attention followed by the CNN module, our module has the ability to learn more information for feature extraction and achieve model interpretability.

#### 2.3.4 Bidirectional long short-term memory

To model sequential data and learn long-range dependencies from protein sequences, we applied a bi-LSTM module in DeepCellEss. LSTM is a type of recurrent neural network that can efficiently mitigate vanishing gradient and exploding gradient issues during long sequence training (Hochreiter and Schmidhuber, 1997). We used a bi-LSTM that can sequentially update the hidden states $H^{\mathrm{lstm}}\in R^{L\times d_{h}}$ for sequential data from two directions, where $d_{h}$ represents the dimension of hidden state vectors. More specifically, let $H_{i}^{\mathrm{lstm}}\in R^{d_{h}}$ denote the hidden state vector of the *i*th residue, which can be formulated by the following equations:

(3) $$\begin{array}{c} I_{i}=\mathrm{sigmoid}\left(H_{i}^{\mathrm{attn}}W^{XI}+H_{i-1}^{\mathrm{lstm}}W^{HI}+b^{I}\right) \end{array}$$

(4) $$\begin{array}{c} F_{i}=\mathrm{sigmoid}\left(H_{i}^{\mathrm{attn}}W^{XF}+H_{i-1}^{\mathrm{lstm}}W^{HF}+b^{F}\right) \end{array}$$

(5) $$\begin{array}{c} O_{i}=\mathrm{sigmoid}\left(H_{i}^{\mathrm{attn}}W^{XO}+H_{i-1}^{\mathrm{lstm}}W^{HO}+b^{O}\right) \end{array}$$

(6) $$\begin{array}{c} C_{i}=F_{i}⊙C_{i-1}+I_{i}⊙\tanh\left(H_{i}^{\mathrm{attn}}W^{XG}+H_{i-1}^{\mathrm{lstm}}W^{HG}+b^{G}\right) \end{array}$$

(7) $$\begin{array}{c} H_{i}^{\mathrm{lstm}}=O_{i}⊙\tanh\left(C_{i}\right) \end{array}$$

where $I_{i},F_{i},O_{i},C_{i}\in R^{L\times d_{h}}$ represents three gates and the cell state at position i of input sequence, respectively, ⊙ stands for the Hadamard product operation, sigmoid$\left(\cdot \right)$ and $\tanh\left(\cdot \right)$ are two types of activation functions. After the bi-LSTM, we obtained the hidden states $H^{\mathrm{lstm}}\in R^{L\times {2d}_{h}}$ as output features by concatenating the hidden states of both directions.

#### 2.3.5 Prediction

In the prediction module, we used a max-pooling layer to down-sample the high-level feature representation from bi-LSTM. Then, the outputted features were fed into a fully connected layer, resulting in a prediction score. Finally, we obtained the prediction essential probability for the input sequence using a sigmoid activation function.

### 2.4 Model interpretability

In addition to accurately predicting essential proteins, we would like to explain visually how DeepCellEss makes specific predictions across different cell lines. To achieve model interpretability, we used a residue-level attention score vector from the multi-head self-attention module to represent the contribution of each residue position. Specifically, for the *j*th single-head self-attention, the original attention score matrix $a_{j}\in R^{L\times L}$ can be calculated from the scaled dot-product attention scoring function,

(8) $$\begin{array}{c} a_{j}=\frac{Q_{j}{K_{j}}^{T}}{\sqrt{d_{k}}} \end{array}$$

Then, we obtained an overall attention score matrix $a\in R^{L\times L}\;$by averaging all single-head attention score matrices. The attention score matrix reflects the relations between any two components of input sequential vectors. In order to assign a score to each sequence position for assessing their contribution to prediction results, we need to convert the score matrix to a score vector with the same size of sequence length. Therefore, we averaged a along the second axis, resulting in an attention score vector $e\in R^{L}$ for each input sequence. Additionally, because we trained five models for each cell line dataset from 5-fold cross-validation, we averaged the attention score vectors from five trained models to obtain the final attention score vector. Through the residue-level attention score vector, we are able to interpret prediction results by locating crucial regions from the input sequence.

### 2.5 Baseline methods

The primary goal of DeepCellEss is to predict the essentiality of proteins using only sequence information. To demonstrate the effectiveness of DeepCellEss, we compared it with five sequence-based baseline methods on the independent test set of HCT-116 benchmark dataset. The baseline methods are described as follows:

1. Seringhaus’s: It is a sequence-based method for essential gene prediction proposed by Seringhaus *et al.* (2006). It extracts 14 features from protein sequences using CodonW, TMHMM v2.0 and PA-SUB v2.5. Then, these sequence-derived features are fed into an ensemble machine-learning model for prediction. We implemented this model and trained it on our benchmark dataset. It should be noted that PA-SUB v2.5 is not available now, so we used Hum-mPLoc 3.0 instead, which is a newly developed protein subcellular localization predictor.
2. EP-GBDT: It extracts the pseudo amino acid composition features using PseAAC, and then integrates multiple GBDT base classifiers to predict essentiality. We re-trained and tested EP-GBDT based on the source code provided in the original paper.
3. EP-EDL: It is a deep learning-based model. For a fair comparison of the model structures, we applied the same sequence representation method as DeepCellEss and re-trained EP-EDL based on its source code.
4. Pheg: It uses λ-interval Z curve method to extract features and SVM classifier to predict essentiality. We directly evaluated Pheg on the independent test set with nucleotide sequence as input using its source code.
5. DeepCellEss-nc: Both protein-level and nucleotide-level sequences have been applied for essentiality prediction. To investigate which type of sequence feature performs better under the same model structure, we modified the original DeepCellEss with nucleotide sequence as input and named it DeepCellEss-nc. We re-trained it using the same sequence representation method and model structure.

### 2.6 Implementation details

We used the hold-out method to evaluate the model performances on our benchmark datasets. In previous studies, the division of dataset into training and test sets was usually performed by the stratified splitting strategy based on the ratio of positive and negative samples. However, since the datasets are imbalanced i.e. the number of non-essential proteins is larger than the number of essential proteins, the stratified splitting strategy will result in an imbalanced test set. In such an imbalanced test set, it is difficult to measure the prediction performance for essential proteins. Therefore, we randomly chose 20% of essential proteins with the equal number of non-essential proteins as the independent test set, and the rest samples as the training set. To make the most use of training data, we applied a 5-fold cross-validation method for model training on the training set. Specifically, the training set was divided into five folds. Each fold is used once for validation and four times for training. After training and validation, we obtained five trained classifiers. When predicting the essentiality on the test set or new protein sequences, the output values of the five classifiers are averaged as the final prediction score.

We performed the training procedure with the mini-batch stochastic gradient descent using the Adam optimizer. To take advantage of the mini-batch technique for training, we utilized the truncation and zero-padding techniques to fix the length of sequence features. To avoid overfitting during the training process, an early stop strategy with a patience of 30 epochs was adopted. To alleviate the class-imbalanced training data problem, we adopted weighted binary cross entropy as the loss function. The loss function $L_{\mathrm{WBCE}}$ is defined as

(9) $$\begin{array}{c} L_{\mathrm{WBCE}}=-\frac{1}{m}\sum_{i=1}^{m}\left(wy_{i}\log\left({\hat{y}}_{i}\right)+\left(1-y_{i}\right)\log\left(1-{\hat{y}}_{i}\right)\right) \end{array}$$

where m is the number of training samples, $y_{i}$ and ${\hat{y}}_{i}$ are the true label and predictive score of sample i. The imbalance parameter w is set to the ratio of the number of negative samples to the number of positive samples.

Our models were implemented in PyTorch and Scikit-learn libraries. All training processes were run with an Intel(R) Xeon(R) Gold 5220 CPU @ 2.20 GHz, 256GB memory and a Nvidia GeForce RTX 2080 Ti GPU. The hyper-parameter settings were determined by grid search techniques.

### 2.7 Evaluation metrics

We evaluated our models on the independent test sets of different cell lines. The model performance was assessed by the area under the receiver-operating characteristic curve (AUROC) and the area under precision-recall curve (AUPRC), which can measure the ranking ability for prediction models. It should be noted that AUPRC is more sensitive to the positive samples i.e. essential proteins and thus can provide more comprehensive evaluation.

## 3. Results

### 3.1 Prediction performance on large-scale datasets of different cell lines

**Fig. 4.**
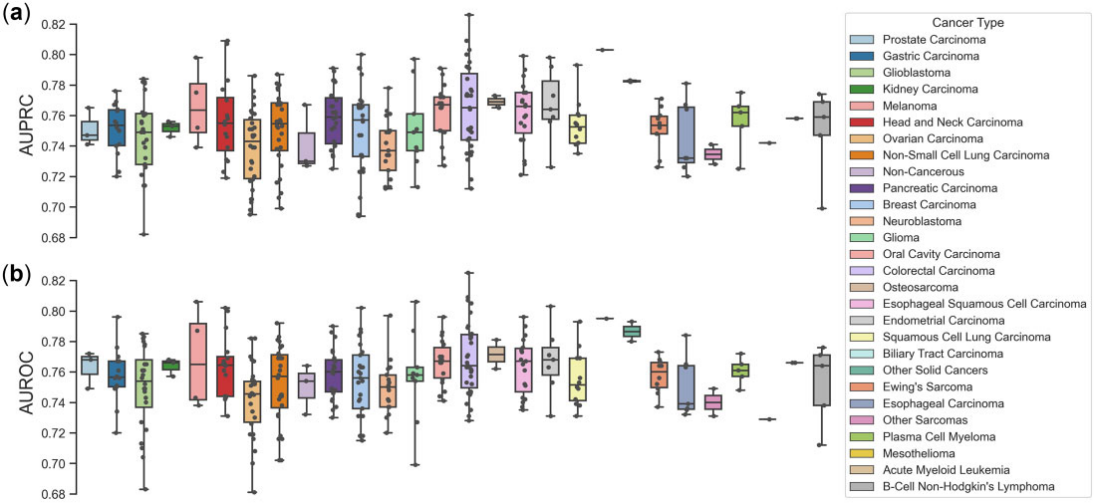
The AUROCs and AUPRCs on the independent test sets of 323 different cell lines using DeepCellEss. (**a**) Boxplots of AUROC (**b**) Boxplots of AUPRC. Since we had 323 cell lines, we classified them into 28 types of cancers and assigned them various colors to represent the 28 types of cancers. Note that the AUROCs, AUPRCs of 323 cell lines mainly vary from 0.72 to 0.80, demonstrating the promising and robust prediction performances of DeepCellEss

To evaluate the performance of DeepCellEss, we trained and tested DeepCellEss on a large collection of benchmark datasets across different cell lines. Specifically, DeepCellEss was trained independently on 323 cell line benchmark datasets using the same model optimization settings. After all training processes are completed, we carried out the tests on the corresponding independent test set of each cell line model. The detailed performance results of all cell line models are listed in Supplementary Table S1. Figure 4 shows them in the form of boxplots. Since we had 323 cell lines, we classified them into 28 groups based on their cancer types and assigned different colors to boxplots for the 28 types of cancers. From Figure 4, we can see that the AUROCs and AUPRCs obtained by DeepCellEss are mainly in the range of 0.72–0.80. Although the performance varies across different cell lines and cancers, the overall prediction performance is robust and promising. In addition, we observed that the best performance is obtained by the SNU-C1 cell line, with an AUROC of 0.825 and an AUPRC of 0.826. The SNU-C1 dataset is a very imbalanced dataset that contains 1298 essential proteins out of a total of 16 408 proteins. The results of SNU-C1 dataset indicate that our model can work well with imbalanced data. Taken together, these results suggest that DeepCellEss is an effective and useful model that can be used for essential protein prediction tasks in various cell lines.

### 3.2 Comparison with baseline methods

In this section, we carried out comparison experiments to investigate the effectiveness of DeepCellEss for essential protein prediction. We compared the performance of DeepCellEss with five sequence-based baseline methods (described in Section 3.4) on the independent test set of HCT-116 cell line. The comparison results are shown in Table 1, which demonstrates that DeepCellEss outperforms the existing sequence-based methods in terms of AUROC and AUPRC. Specifically, when compared to other baseline methods, DeepCellEss achieves AUROC and AUPRC scores of 0.782 and 0.795, with an increase of 1.8–45.4% and 2.3–76.7%, respectively.

**Table 1.** Performances of DeepCellEss and existing sequence-based methods on the independent test set of HCT-116

Method	AUROC	AUPRC
Seringhaus's	0.734	0.682
EP-GBDT	0.768	0.777
EP-EDL	0.760	0.736
Pheg	0.427	0.450
DeepCellEss-nc	0.751	0.740
DeepCellEss	**0.782**	**0.795**

*Note:* The best performance values are highlighted in bold.

In addition, we can see that Pheg gets AUROC and AUPRC scores of 0.427 and 0.450, respectively, which are lower than the other methods. This can be explained by the fact that Pheg web server only provides a general human gene essentiality predictor, and it ignores specific differences in the essentiality of genes and the encoded products across cell lines, resulting in poor prediction performance on cell line-specific test datasets. Such results indicate the difficulty of identifying cell line-specific essential genes and proteins using a general model trained on common essential samples in cell lines. Thus, training cell line-specific models is necessary for discovering specific essential genes and proteins in different cell lines.

Moreover, we observed that the performance of DeepCellEss is better than DeepCellEss-nc, which means that protein sequence features are more effective than nucleotide sequence features for DeepCellEss model. The results may be thanks to that: (i) protein sequences are composed of 21 types of amino acids while nucleotide sequences are made up of four different types of nucleotides, resulting in protein sequences has a more diverse sequence information; (ii) the encoded protein sequence is much shorter than the nucleotide sequence for a gene, which can reduce computational consumption and processing complexity; and (iii) protein sequence features are more informative for essentiality prediction.

### 3.3 Comparison with network-based methods under cell line-specific networks

Over the past two decades, many studies have reported that the essentiality of proteins is highly related to the topological properties of PPI networks. Extensive network-based centrality measures were developed for discovering new essential proteins. These methods can efficiently mine latent information from network topology and rank essentiality for proteins in PPI networks. However, the network-based methods suffer from several major drawbacks: (i) these methods cannot be directly used for proteins that are not in the PPI networks; (ii) their prediction ability for essential proteins with low degrees is greatly limited. As a sequence-based method, DeepCellEss is able to compensate for the shortcomings of network-based centrality measures.

In order to investigate whether DeepCellEss can achieve promising prediction performance without interaction information, we designed the following experiments: We first downloaded protein interaction data of HCT-116 cell line from the BioPlex 3.0 database (Huttlin *et al.*, 2021) and constructed an HCT-116 cell line-specific PPI network (referred to as the HCT-116 network), which includes 10 115 proteins and 70 966 interactions in total. Then, six classical network-based centrality measures i.e. BC, CC, DC, EC, LAC and NC, are calculated for all 10 115 protein nodes in the HCT-116 network. The scores of BC, CC, DC and EC are calculated using the python library NetworkX (Hagberg *et al.*, 2008) and the scores of LAC (Li *et al.*, 2011) and NC (Wang *et al.*, 2012) are calculated based on the proposed methods.

**Fig. 5.**
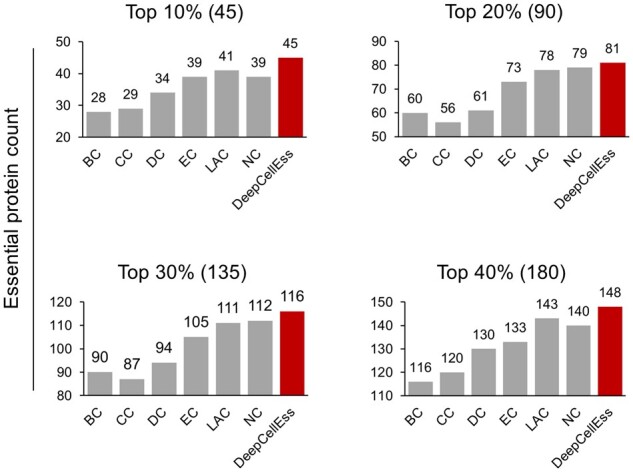
Count of essential proteins detected by network-based methods and DeepCellEss based on the ranked prediction scores on the new HCT-116 test set

To compare the performance of DeepCellEss with network-based centrality measures based on the same dataset, we screened the 450 intersection proteins of HCT-116 network and HCT-116 test set as a new test set, which includes equal numbers (225) of essential and non-essential proteins. We ranked the result scores predicted by the six network-based methods and DeepCellEss from highest to lowest and compared the cumulative counts of essential proteins in the top 10%, top 20%, 30% and top 40% proteins. The results in Figure 5 show that DeepCellEss is able to identify more essential proteins than centrality measure methods.

We further explored the prediction performance of DeepCellEss on the proteins with low degree in PPI networks. According to the Centrality-Lethality Rule (Jeong *et al.*, 2001), higher centrality measure values indicate higher essentiality of proteins. Therefore, network-based methods usually predict the proteins with low degree to be non-essential, resulting they could barely identify those essential proteins that have few interaction partners or lack of interaction information. To evaluate how well DeepCellEss performs on the low-degree essential proteins, we screened the 147 essential proteins with only one degree in the HCT-116 network. Then, we re-split the HCT-116 dataset with these 147 essential proteins as the new independent test set and the rest as the new training set. After re-training the HCT-116-specific DeepCellEss model, the results show that 69.4% (102) of the 147 essential proteins could be accurately predicted, indicating that our model has practical and effective prediction ability for the essential proteins on the low-degree essential proteins.

To better illustrate the prediction performance of different types of methods for the proteins with low degree, we gave prediction results of an example essential protein ‘Probable ATP-dependent RNA helicase DDX59’ (DDX59, Uniprot ID: Q5T1V6). DDX59 is a member of the DEAD box helicase family proteins, which involves in all aspects of RNA metabolism and plays an important role in many cellular activities. Supplementary Figure S2 shows the local connectivity information of DDX59. From the ranking results of the HCT-116 network using six classical centrality measure scores [i.e. DC, BC, CC, EC, NC, LAC), DDX59 ranked 9097 (89.9%), 9033 (89.3%), 9671 (95.6%), 8920 (88.2%), 9783 (96.7%) and 9783 (96.7%) out of 10 115 proteins, respectively]. These network-based methods cannot identify the cell line-specific essentiality of DDX59, while DeepCellEss predicts DDX59 correctly with the essentiality score of 0.788 in the HCT-116-specific model.

Last, we explored the performance of DeepCellEss on the proteins that have no protein interaction information in our HCT-116 benchmark dataset. Based on our statistics, there are 438 essential proteins that do not appear in the HCT-110 network, which means they are not able to be identified by network-based approaches. Then, we tested them using our re-trained HCT-116-specific model. The results show that 63.9% (280) of them are correctly predicted, indicating that our model has practical and effective prediction ability for essential protein prediction without PPI information.

Overall, these experiment results and comparative analysis of DeepCellEss and network-based methods confirm that DeepCellEss can achieve promising performance for essential proteins with no PPI information or low degree in PPI networks. DeepCellEss effectively compensates for the limitations of network-based methods and offers a more practical approach to essential protein prediction.

### 3.4 Case studies

**Fig. 6.**
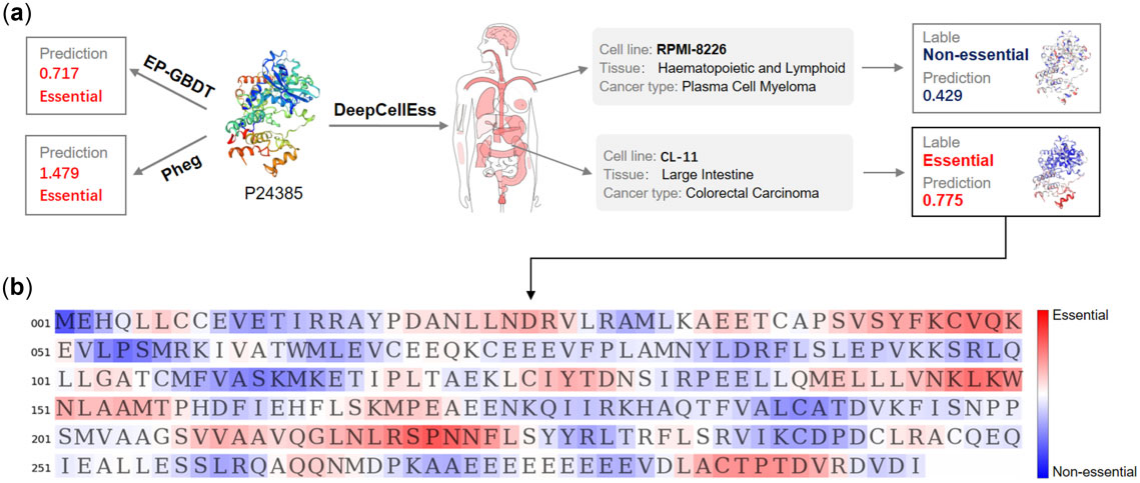
Case study for cell line-specific predictions and model interpretability by DeepCellEss on CCND1. (**a**) Predictions of CCND1 (Uniprot ID: P24385) by three available online predictors. DeepCellEss enables accurate cell line-specific predictions while Pheg and EP-GBDT only give a unified result for all cell lines. (**b**) Interpretability for the prediction of P24385 in CL-11. In the visual heatmap, the red regions indicate higher attention scores that contribute more to essential, and the blue regions indicate lower attention scores that contribute more to non-essential

A major advantage of our proposed model is the capability to learn and predict protein essentialities across different cell lines. To demonstrate the effectiveness of our model for cell line-specific prediction, we used ‘G1/S-specific cyclin-D1’ (CCND1, Uniprot ID: P24385) as an example to compar DeepCellEss with other two available servers (Pheg and EP-GBDT). From pre-existing biological experiments, CCND1 performs obvious differences within various cellular environments in terms of essentiality. For instance, it is identified as essential in CL-11 cell line while non-essential in RPMI-B226 cell line. Figure 6a presents the prediction results of CCND1 using DeepCellEss, Pheg and EP-GBDT, respectively. With the support of cell line-specific predictions, DeepCellEss gets different essentiality scores with 0.429 of RPMI-B226 and 0.775 of CL-11, yielding accurate predictions of CCND1 under different cell lines. However, Pheg and EP-GBDT can only give overall prediction scores of 0.717 and 1.479 because they cannot support cell line-level prediction. Both Pheg and EP-GBDT predict CCND1 as an essential protein but fail to capture the non-essentiality of CCND1 in cell line of RPMI-B226.

Moreover, DeepCellEss leverages the advantage of the attention mechanism to assign residue-level attention scores for query proteins, and provides the visual heatmap for interpretation. Figure 6b shows the prediction heatmap of CCND1 in CL-11. The red regions indicate contributions to be essential while the blue regions indicate contributions to be non-essential in the prediction. To further illustrate our interpretable model is possible to detect regions which are important motifs and correlated with protein essential functions, we performed a case study as follows:

The JAB1/MPN/Mov34 metalloenzyme (JAMM) motif is highly conserved, typically consisting of a canonical sequence of ‘H-[NST]-H-x(7)-S-x(2)-D’. JAMM-containing proteins are metal-dependent proteases and responsible for providing the active site for isopeptidase activity (Ambroggio *et al.*, 2004). Supplementary Figure S3a shows the JAMM motif logo generated from JAMM-containing proteins in UniprotKB database using MEME (Bailey *et al.*, 2015). PSMD14/Rpn11/POH1 is a representative JAMM-containing protein. PSMD14 plays a key role within the proteasomes, where it acts as an intrinsic deubiquitinase removing polyubiquitin chains from substrate proteins (Wauer and Komander, 2014). Research evidence suggests that the JAMM motif of SMD14 is essential for human cell viability (Gallery *et al.*, 2007; Verma *et al.*, 2002). We used DeepCellEss to predict PSMD14 (Uniport ID: O00487) under ‘Unknown’ cancer type and ‘Unknown’ cell line options. Supplementary Figure S3b shows the prediction result (0.687) and the visualization heatmap of SMD14. In the heatmap, the JAMM motif is marked red in the whole sequence. The results suggest that our predictor could identify essential protein and might recognize its important motif.

Additionally, we analyzed the performance of DeepCellEss on intrinsically disordered proteins (IDPs), which are widely distributed in eukaryotes and closely associated with human diseases. From the cancer-related protein dataset of DisProt database, we found a conditional essential IDP with 100% disorder content, called ‘nuclear factor erythroid 2-related factor 2’ (NFE2L2, Uniprot ID: Q16236). Several studies have revealed that NFE2L2 is highly related to lung cancers (Binkley *et al.*, 2020; Sánchez-Ortega *et al.*, 2021). We used DeepCellEss to predict NFE2L2 under the options of ‘Non-Small Cell Lung Carcinoma’, ‘Squamous Cell Lung Carcinoma’, and ‘No-cancerous’ cancer types, respectively. The results (shown in Supplementary Fig. S4) indicate that DeepCellEss predicts NFE2L2 to be essential in two types of lung cancers but non-essential in non-cancerous, implying that our essentiality predictor is useful for IDPs and has the potential to find some cancer-related essential IDPs.

### 3.5 Ablation study

To measure the contributions of individual components to DeepCellEss structure, we conducted ablation studies by re-training and validating DeepCellEss without different components. Specifically, four main components, including skip connection, CNN module, multi-head self-attention module and bi-LSTM module, were separately removed, and we obtained four variants of DeepCellEss model. Then, we trained and validated these four models. The strategies for dataset splitting and model training remain unchanged as the raw DeepCellEss. Table 2 reports the results of DeepCellEss and its variants, which show that the removal of the different components leads to a reduction in the prediction performance of DeepCellEss. Our model yields the best AUROC of 0.782 and the best AUPRC of 0.795, in which the AUPRC is improved by about 4.1%, 2.2%, 4.2% and 3.7% over DeCepCellEss without skip connection, CNN module, multi-head self-attention module and bi-LSTM module, respectively. The ablation studies demonstrate that the model architecture of raw DeepCellEss is optimal for our prediction task.

**Table 2.** The performances of DeepCellEss and its variant models in the ablation study

Model	AUROC	AUPRC
Without skip connection	0.759	0.764
Without CNN module	0.765	0.776
Without multi-head self-attention module	0.766	0.763
Without bi-LSTM module	0.754	0.767
DeepCellEss	**0.782**	**0.795**

*Note:* The best performance values are highlighted in bold.

### 3.6 Web server

To facilitate the access to DeepCellEss, we developed a user-friendly web server, http://csuligroup.com:8000/DeepCellEss. The DeepCellEss web server provides cell line-specific essential protein prediction and visualization for a large amount of cell lines. Supplementary Figure S5 shows the user interface of DeepCellEss web server. Users can enter an UniProt ID to search for the protein sequence or directly input a single protein sequence with length less than 1000aa in FASTA format, and then choose a cell line from a list of 323 cell lines, to predict and analyze protein essentiality in the certain cell line environment. Besides, if users are unsure which cell line environment the query protein is located in, we offer an option of ‘Unknown’ to enable a unified result for human protein essentiality prediction. The results of this option are the average prediction score under all cell line-specific models. For each submission, the output panel presents two parts, i.e., the result of predicted essentiality and the visualization of residue-level attention scores. The result part gives a five-column table containing the cell line name, the input protein ID, the sequence length, the predicted essentiality score and the final predicted label. The visualization part provides a heatmap and an interactive line plot, which allows users to estimate the contribution of each residue position to the prediction results from various perspectives. To the best of our knowledge, it is the first web server that can predict essential proteins under specific cell lines and provide visualization analysis. We believe that DeepCellEss can serve as a practical and useful tool for human essential protein study.

## 4. Conclusion

The identification of cancer cell line-specific essential proteins is particularly relevant for the discovery of novel precision cancer drug targets. However, existing computational methods have not taken into account the specificity of essential proteins in different cell lines, and lack practical and interpretable tools for human essential protein prediction. In this study, we proposed DeepCellEss, a cell line-specific interpretable deep learning prediction method based on the attention mechanism. The main contributions of DeepCellEss are summarized as follows:

- To the best of our knowledge, DeepCellEss is the first computational method that supports cell line-specific essential protein predictions, which makes it possible to predict protein essentialities in different cellular environments;
- DeepCellEss implements an interpretable deep-learning model through residue-level attention scores from multi-head self-attention mechanism. The attention scores enable to locate the most important sequence regions for different prediction results, and further make more comprehensive analysis and comparison for cell line-specific essential proteins;
- For real practical applications of our cell line-specific model, we constructed extremely large-scale datasets across 323 cell lines. Moreover, we provided a user-friendly web server of cell line-specific essential protein predictions. It is expected to help discover potential diagnostic biomarkers and therapeutic targets for precision cancer therapy.

Although the extensive results show that DeepCellEss is an effective predictor for cell line-specific essential proteins and outperformers existing sequence-based methods, we would like to point out its limitations. The main limitation is that we do not consider the relations of different cell lines under the same tissue or cancer type. In our reported results, the models of different cell lines under the same cancer type show varying prediction performance. For example, in the cancer type of Colorectal Carcinoma, SNU-C1 model yields the best AUROC (0.825) and AUPRC (0.826), while MDST8 model gets the worst AUROC (0.728) and AUPRC (0.731). Therefore, future efforts could be devoted to improving the poor performance for some cell lines by introducing the relations between different cell lines. One potential solution is to use transfer learning techniques (Pan and Yang, 2010; Zeng *et al.*, 2019). To be specific, we can first pre-train with multiple cell line datasets that are closely related to the target cell line, and then apply the knowledge to the target cell line dataset to develop a more powerful cell line-specific model.

## Supplementary Material

btac779_Supplementary_Data
