# Supplementary material for: DeepCellEss: cell line-specific essential protein prediction with attention-based interpretable deep learning
Source: Bioinformatics. 2022 Dec 2;39(1):btac779. doi: 10.1093/bioinformatics/btac779 (PMC9825760; doi:10.1093/bioinformatics/btac779)
Supplement: btac779_Supplementary_Data [file btac779_supplementary_data.pdf]

Supplementary materials:

DeepCellEss: Cell line-specific essential protein prediction with attention-based interpretable deep learning

Yiming Li[1], Min Zeng[1], Fuhao Zhang[1], Fang-Xiang Wu[2], and Min Li[1,*]

[1]Hunan Provincial Key Lab on Bioinformatics, School of Computer Science and Engineering, Central South University, Changsha, 410083, China.

[2]Division of Biomedical Engineering, Department of Computer Science, Department of Mechanical Engineering University of Saskatchewan Saskatoon, SK S7N 5A9, Canada.

Contact: limin@mail.csu.edu.cn

The supplementary file includes:

1. Supplementary Figures

Fig. S1. Heatmaps of pairwise Pearson correlation coefficients for essential protein data across cell lines of different cancer types.

Fig. S2. The local connectivity information of DDX59.

Fig. S3. JAMM motif and prediction results for PSMD14 using DeepCellEss.

Fig. S4. Prediction results of NFE2L2 using DeepCellEss.

Fig. S5. User interface of DeepCellEss web server for cell line-specific essential protein prediction.

2. Supplementary Tables

Table S1. The AUROC and AUPRC of DeepCellEss on the independent test sets of 323 cell lines

1. Supplementary Figures

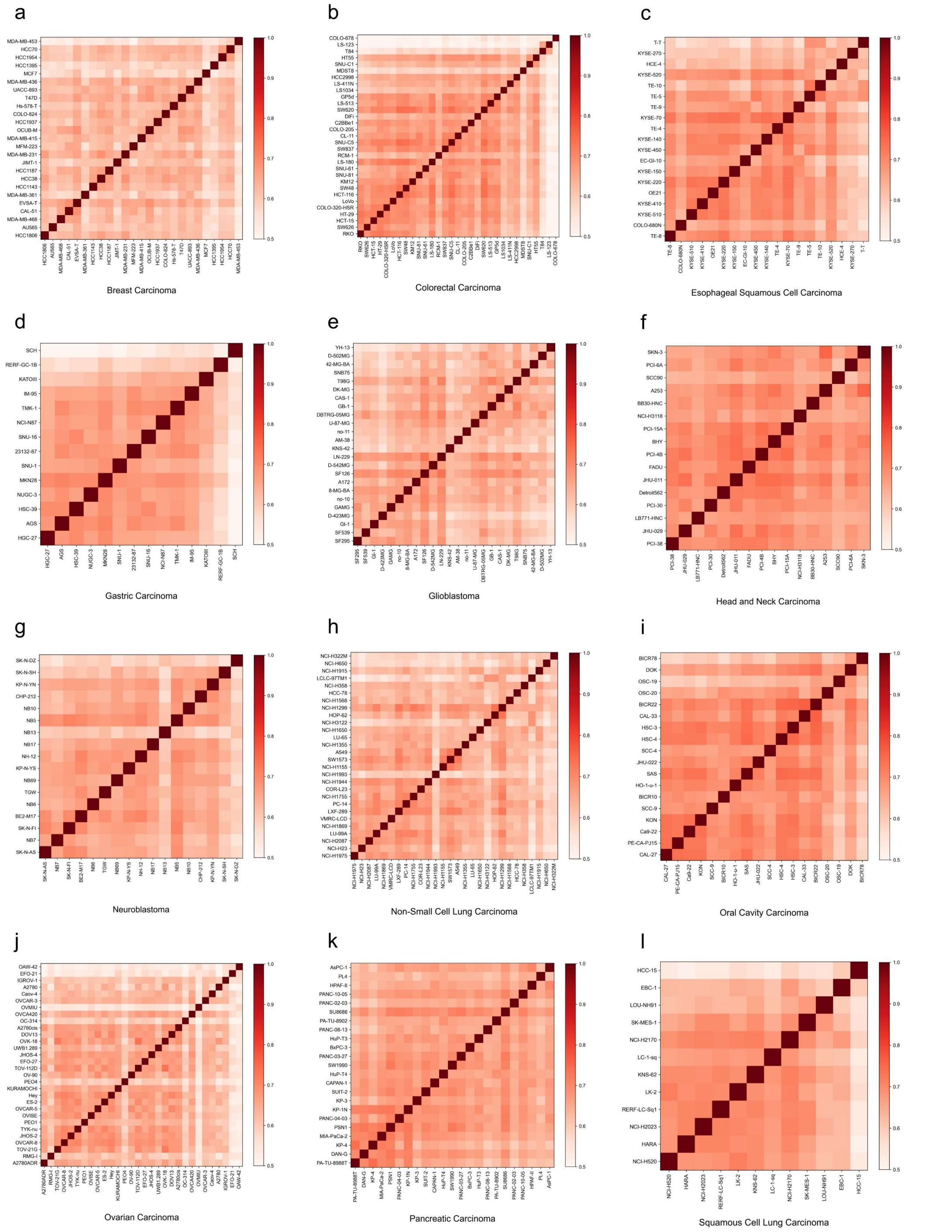

Fig. S1. Heatmaps of pairwise Pearson correlation coefficients for essential protein data across cell lines of different cancer types. (a) Breast carcinoma. (b) Esophageal squamous cell carcinoma. (c) Gastric carcinoma. (d) Glioblastoma. (e) Head and neck carcinoma. (f) Neuroblastoma. (g) No-small cell lung carcinoma. (h) Oral cavity carcinoma. (i) Ovarian carcinoma. (j) Pancreatic carcinoma. (k) Squamous cell lung carcinoma.

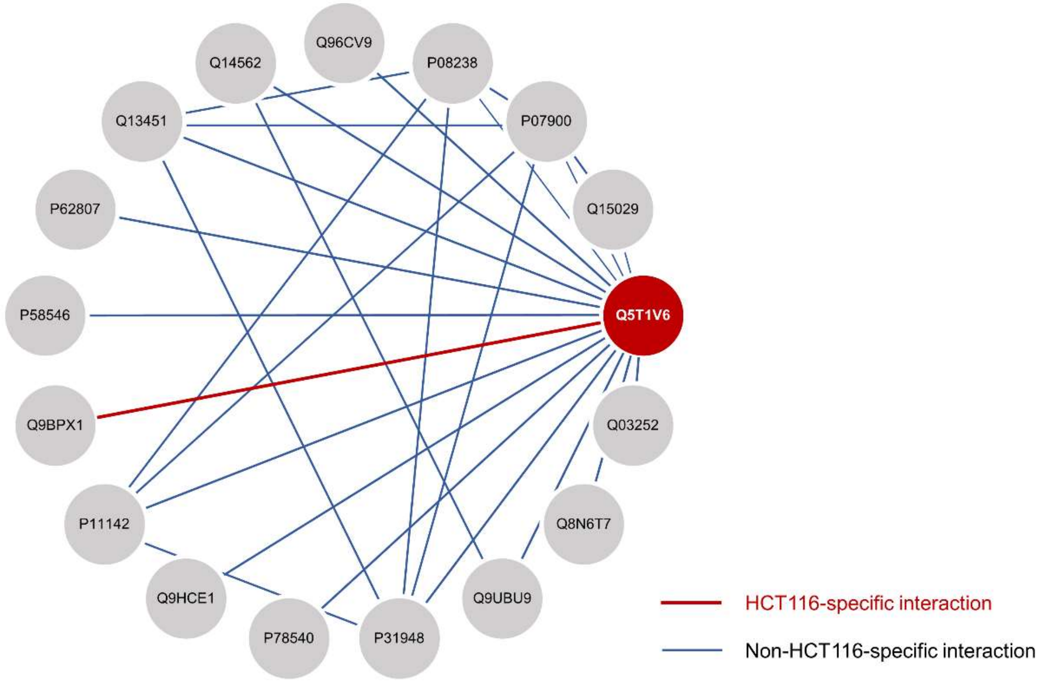

Fig. S2. The local connectivity information of DDX59 (Uniprot ID: Q5T1V6). Red lines represent the interactions existing in HCT 116 cell line, blue lines represent the interactions existing in other cell lines.

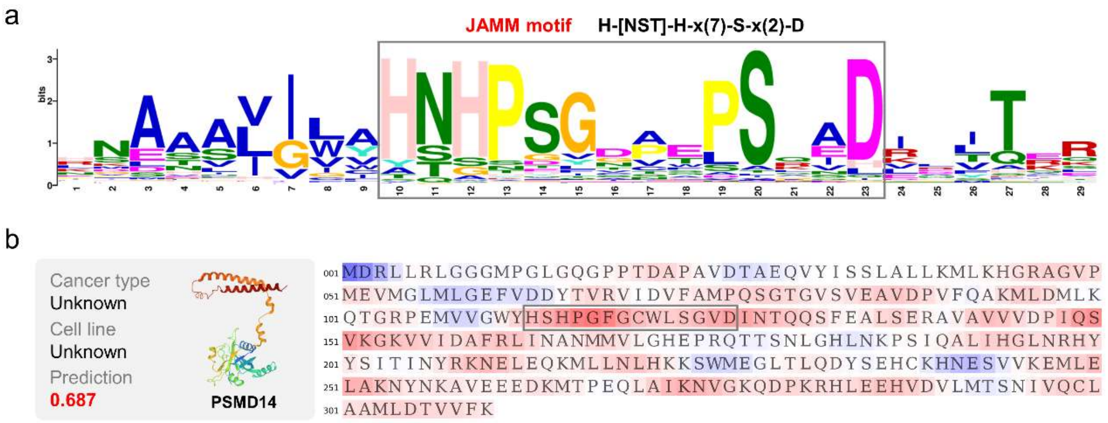

Fig. S4. JAMM motif and prediction results for PSMD14 (Uniport ID: O00487) using DeepCellEss. (a) The JAMM motif logo generated from JAMM-containing proteins in UniprotKB database using the MEME tool. (b) Visualization heatmap of O00487 under “Unknown” cancer type and “Unknown” cell line option. The functional conserved motif JAMM (marked by grey boxes) is detected and highlighted by DeepCellEss.

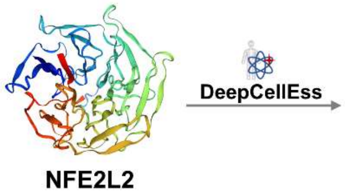

Cancer type	Cell line	Prediction
Non-Small Cell Lung Carcinoma	Unknown	0.535
Squamous Cell Lung Carcinoma	Unknown	0.559
Non-cancerous	Unknown	0.428

Fig. S5. Prediction results of NFE2L2 (Uniport ID: Q16236) using DeepCellEss. The cancer type options are set to "Non-Small Cell Lung Carcinoma", "Squamous Cell Lung Carcinoma", and "No-cancerous", respectively. The cell line options are set to "Unknown".

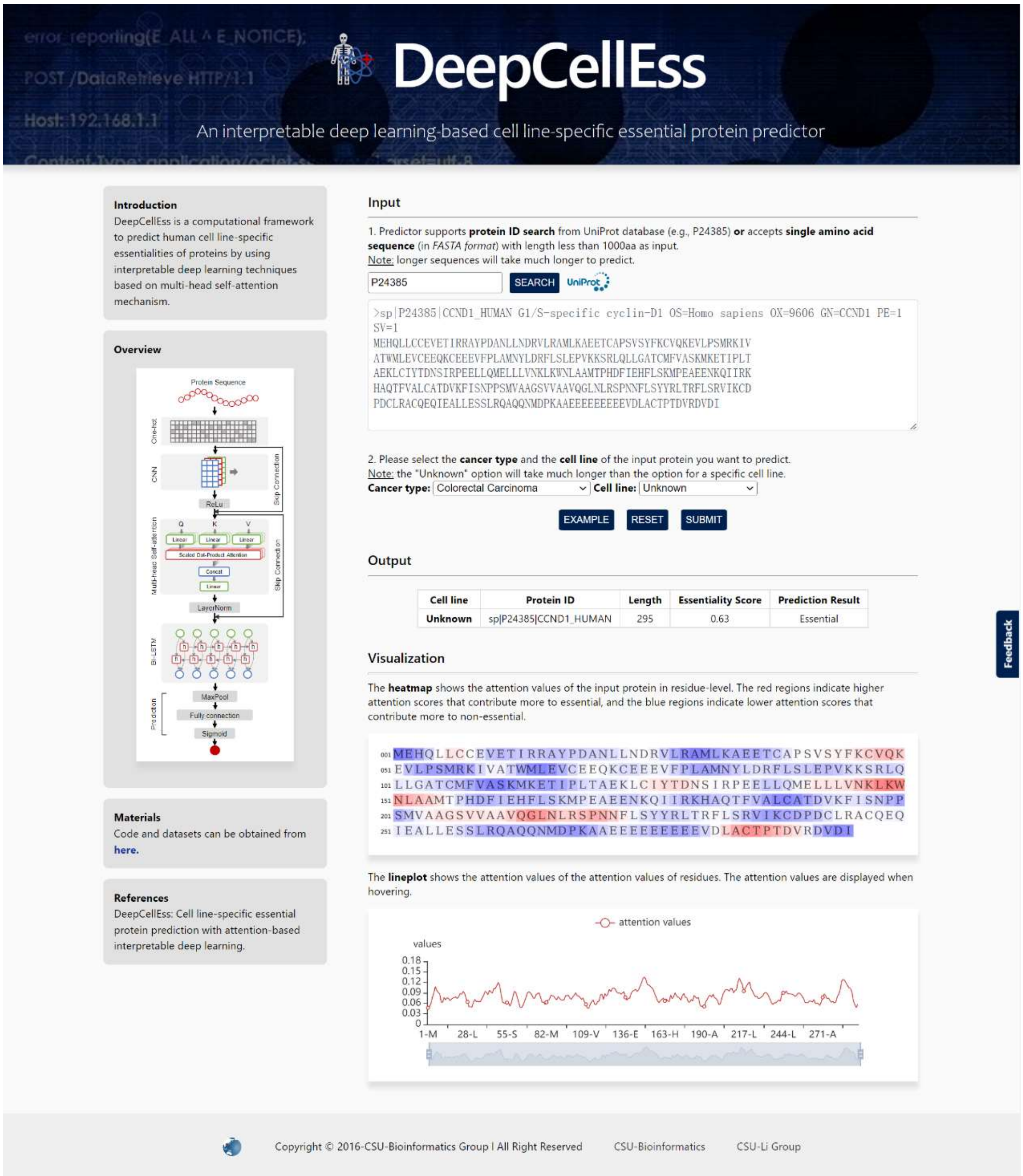

Fig. S6. User interface of DeepCellEss web server for cell line-specific essential protein prediction.

2. Supplementary Tables

Table S1. The AUROC and AUPRC of independent test sets for 323 cell lines

	Cell line	AUROC	AUPRC		Cell line	AUROC	AUPRC
1	22RV1	0.749	0.747	43	COLO-824	0.786	0.787
2	23132-87	0.767	0.752	44	COR-L23	0.769	0.767
3	42-MG-BA	0.761	0.755	45	D-423MG	0.713	0.728
4	769-P	0.757	0.746	46	D-502MG	0.785	0.782
5	8-MG-BA	0.777	0.782	47	D-542MG	0.761	0.756
6	A172	0.756	0.727	48	DAN-G	0.746	0.74
7	A2058	0.743	0.739	49	DBTRG-05MG	0.74	0.732
8	A253	0.744	0.731	50	Detroit562	0.744	0.723
9	A2780	0.752	0.751	51	DiFi	0.731	0.734
10	A2780ADR	0.752	0.746	52	DK-MG	0.722	0.714
11	A2780cis	0.77	0.772	53	DOK	0.78	0.787
12	A375	0.787	0.775	54	DOV13	0.745	0.718
13	A549	0.745	0.753	55	DU-145	0.772	0.765
14	AGS	0.751	0.756	56	EBC-1	0.739	0.746
15	AM-38	0.683	0.682	57	EC-GI-10	0.737	0.728
16	ARH-77	0.754	0.73	58	EFO-21	0.7	0.703
17	AsPC-1	0.73	0.725	59	EFO-27	0.752	0.747
18	AU565	0.718	0.723	60	EGI-1	0.795	0.803
19	BB30-HNC	0.802	0.809	61	EPLC-272H	0.78	0.782
20	BE2-M17	0.741	0.734	62	ES-2	0.745	0.751
21	Becker	0.756	0.749	63	ES4	0.756	0.755
22	BHY	0.761	0.739	64	ES5	0.737	0.748
23	BICR10	0.766	0.772	65	ES8	0.767	0.766
24	BICR22	0.768	0.765	66	ESO26	0.735	0.72
25	BICR78	0.768	0.766	67	ESO51	0.739	0.726
26	BxPC-3	0.742	0.743	68	ESS-1	0.749	0.741
27	C2BBe1	0.805	0.803	69	EVSA-T	0.778	0.769
28	Ca9-22	0.744	0.732	70	EW-1	0.768	0.758
29	CAL-27	0.766	0.768	71	EW-16	0.746	0.748
30	CAL-33	0.77	0.749	72	EW-22	0.773	0.771
31	CAL-51	0.745	0.725	73	EW-7	0.764	0.752
32	CAL-72	0.781	0.773	74	FADU	0.789	0.777
33	Caov-4	0.746	0.74	75	FLO-1	0.736	0.732
34	CAPAN-1	0.759	0.751	76	GAMG	0.71	0.714
35	CAS-1	0.765	0.762	77	GB-1	0.783	0.777
36	CHP-212	0.768	0.763	78	GI-1	0.758	0.749
37	CL-11	0.766	0.776	79	GP5d	0.809	0.8
38	COLO-205	0.757	0.744	80	HARA	0.769	0.767
39	COLO-320-HSR	0.77	0.765	81	HCC-15	0.769	0.76
40	COLO-678	0.771	0.773	82	HCC-78	0.718	0.706
41	COLO-680N	0.735	0.73	83	HCC1143	0.759	0.747
42	COLO-684	0.781	0.792	84	HCC1187	0.76	0.76

	Cell line	AUROC	AUPRC		Cell line	AUROC	AUPRC
85	HCC1395	0.754	0.765	131	KP-4	0.748	0.737
86	HCC1806	0.756	0.756	132	KP-N-YN	0.797	0.778
87	HCC1937	0.715	0.695	133	KP-N-YS	0.753	0.724
88	HCC1954	0.773	0.77	134	KURAMOCHI	0.719	0.705
89	HCC2998	0.76	0.718	135	KYAE-1	0.765	0.767
90	HCC38	0.764	0.757	136	KYSE-140	0.796	0.79
91	HCC70	0.784	0.791	137	KYSE-150	0.776	0.779
92	HCE-4	0.774	0.775	138	KYSE-220	0.773	0.775
93	HCT-116	0.782	0.795	139	KYSE-270	0.74	0.744
94	HCT-15	0.752	0.74	140	KYSE-410	0.752	0.77
95	HEC-1	0.753	0.756	141	KYSE-450	0.742	0.721
96	Hey	0.745	0.747	142	KYSE-510	0.751	0.744
97	HGC-27	0.756	0.75	143	KYSE-520	0.768	0.759
98	HO-1-u-1	0.741	0.739	144	KYSE-70	0.775	0.766
99	HOP-62	0.792	0.781	145	L-363	0.757	0.753
100	HPAF-II	0.763	0.783	146	LB1047-RCC	0.766	0.753
101	Hs-578-T	0.741	0.734	147	LB771-HNC	0.8	0.807
102	Hs-683	0.727	0.73	148	LC-1-sq	0.738	0.741
103	HSC-3	0.786	0.78	149	LCLC-97TM1	0.764	0.769
104	HSC-39	0.756	0.735	150	LK-2	0.749	0.751
105	HSC-4	0.784	0.772	151	LN-229	0.765	0.751
106	HT-29	0.746	0.745	152	LNCaP-Clone-FGC	0.768	0.741
107	HT55	0.762	0.752	153	LNZTA3WT4	0.758	0.737
108	hTERT-RPE-1	0.764	0.767	154	LOU-NH91	0.75	0.755
109	HuP-T3	0.769	0.758	155	LoVo	0.764	0.774
110	HuP-T4	0.783	0.762	156	LP-1	0.772	0.775
111	IGROV-1	0.739	0.729	157	LS-123	0.79	0.809
112	IM-9	0.732	0.727	158	LS-180	0.746	0.749
113	IM-95	0.767	0.769	159	LS-411N	0.739	0.736
114	IST-MEL1	0.738	0.752	160	LS-513	0.747	0.744
115	JHOS-2	0.719	0.698	161	LS1034	0.785	0.784
116	JHOS-4	0.755	0.752	162	LU-65	0.716	0.716
117	JHU-011	0.769	0.754	163	LU-99A	0.758	0.753
118	JHU-022	0.778	0.768	164	LXF-289	0.783	0.787
119	JHU-029	0.767	0.77	165	MC-IXC	0.752	0.73
120	JIMT-1	0.753	0.766	166	MCF7	0.736	0.694
121	KATOIII	0.75	0.763	167	MDA-MB-231	0.731	0.733
122	KINGS-1	0.763	0.761	168	MDA-MB-361	0.718	0.734
123	KLE	0.803	0.798	169	MDA-MB-415	0.771	0.765
124	KM12	0.789	0.791	170	MDA-MB-436	0.731	0.723
125	KMS-11	0.748	0.725	171	MDA-MB-453	0.763	0.767
126	KNS-42	0.704	0.726	172	MDA-MB-468	0.715	0.706
127	KNS-62	0.793	0.793	173	MDST8	0.728	0.731
128	KON	0.755	0.75	174	MFE-280	0.768	0.764
129	KP-1N	0.76	0.752	175	MFE-296	0.763	0.759
130	KP-3	0.764	0.782	176	MFM-223	0.763	0.766

	Cell line	AUROC	AUPRC		Cell line	AUROC	AUPRC
177	MHH-ES-1	0.749	0.726	223	OE21	0.767	0.76
178	MIA-PaCa-2	0.749	0.755	224	OE33	0.784	0.781
179	MKN28	0.734	0.72	225	OPM-2	0.761	0.766
180	MOG-G-UVW	0.759	0.751	226	OSC-19	0.77	0.768
181	NB10	0.742	0.73	227	OSC-20	0.796	0.791
182	NB13	0.72	0.713	228	OV-90	0.751	0.739
183	NB17	0.748	0.75	229	OVCA420	0.782	0.776
184	NB5	0.763	0.76	230	OVCAR-3	0.754	0.761
185	NB6	0.758	0.761	231	OVCAR-5	0.782	0.786
186	NB69	0.751	0.749	232	OVCAR-8	0.767	0.762
187	NB7	0.75	0.737	233	OVISE	0.726	0.725
188	NCI-H1155	0.769	0.768	234	OVK-18	0.768	0.767
189	NCI-H1299	0.776	0.778	235	OVMIU	0.708	0.705
190	NCI-H1355	0.78	0.767	236	PA-TU-8902	0.762	0.77
191	NCI-H1568	0.738	0.748	237	PA-TU-8988T	0.763	0.766
192	NCI-H1650	0.771	0.757	238	PANC-02-03	0.773	0.773
193	NCI-H1755	0.777	0.756	239	PANC-03-27	0.735	0.74
194	NCI-H1869	0.702	0.699	240	PANC-04-03	0.767	0.762
195	NCI-H1915	0.772	0.777	241	PANC-08-13	0.737	0.733
196	NCI-H1944	0.744	0.727	242	PANC-10-05	0.748	0.759
197	NCI-H1975	0.731	0.752	243	PC-14	0.744	0.738
198	NCI-H1993	0.732	0.734	244	PCI-15A	0.773	0.783
199	NCI-H2023	0.777	0.761	245	PCI-30	0.731	0.719
200	NCI-H2087	0.702	0.718	246	PCI-38	0.765	0.76
201	NCI-H2170	0.756	0.741	247	PCI-4B	0.765	0.758
202	NCI-H226	0.729	0.742	248	PCI-6A	0.764	0.756
203	NCI-H23	0.769	0.78	249	PE-CA-PJ15	0.747	0.727
204	NCI-H3118	0.742	0.73	250	PEO1	0.716	0.711
205	NCI-H3122	0.742	0.739	251	PEO4	0.734	0.73
206	NCI-H322M	0.761	0.764	252	PL4	0.741	0.734
207	NCI-H358	0.745	0.744	253	PSN1	0.79	0.791
208	NCI-H520	0.742	0.742	254	RCC-FG2	0.768	0.756
209	NCI-H650	0.716	0.709	255	RCM-1	0.753	0.75
210	NCI-N87	0.796	0.776	256	RERF-GC-1B	0.72	0.723
211	NH-12	0.737	0.718	257	RERF-LC-Sq1	0.731	0.735
212	NMC-G1	0.806	0.797	258	RKN	0.731	0.728
213	no-10	0.747	0.744	259	RKO	0.783	0.764
214	no-11	0.743	0.742	260	RL95-2	0.771	0.773
215	NUGC-3	0.761	0.755	261	RMG-I	0.681	0.695
216	OACM5-1	0.732	0.732	262	ROS-50	0.738	0.747
217	OAW-42	0.719	0.72	263	RPMI-8226	0.765	0.762
218	OC-314	0.73	0.717	264	SAS	0.766	0.765
219	OCI-AML2	0.766	0.758	265	SCC-4	0.753	0.75
220	OCI-AML3	0.766	0.758	266	SCC-9	0.759	0.774
221	OCI-LY-19	0.776	0.774	267	SCC90	0.743	0.748
222	OCUB-M	0.802	0.8	268	SCH	0.749	0.74

	Cell line	AUROC	AUPRC		Cell line	AUROC	AUPRC
269	SF126	0.78	0.784	297	SW1573	0.781	0.771
270	SF295	0.727	0.721	298	SW1990	0.786	0.789
271	SF539	0.749	0.739	299	SW48	0.761	0.773
272	SJSA-1	0.762	0.765	300	SW620	0.763	0.751
273	SK-GT-4	0.763	0.764	301	SW626	0.735	0.712
274	SK-MEL-2	0.806	0.798	302	SW837	0.779	0.773
275	SK-MES-1	0.753	0.754	303	T-T	0.781	0.786
276	SK-MG-1	0.754	0.739	304	T47D	0.788	0.791
277	SK-N-AS	0.755	0.749	305	T84	0.799	0.792
278	SK-N-DZ	0.737	0.742	306	T98G	0.782	0.76
279	SK-N-FI	0.732	0.712	307	TC-71	0.764	0.76
280	SK-N-SH	0.73	0.713	308	TE-10	0.784	0.769
281	SK-PN-DW	0.793	0.783	309	TE-4	0.765	0.755
282	SKN-3	0.758	0.746	310	TE-5	0.79	0.799
283	SNB75	0.749	0.761	311	TE-8	0.743	0.753
284	SNG-M	0.731	0.726	312	TE-9	0.761	0.767
285	SNU-1	0.757	0.741	313	TGW	0.762	0.734
286	SNU-16	0.77	0.767	314	TMK-1	0.776	0.764
287	SNU-61	0.784	0.777	315	TOV-112D	0.752	0.763
288	SNU-81	0.745	0.735	316	TOV-21G	0.753	0.755
289	SNU-C1	0.825	0.826	317	TYK-nu	0.744	0.739
290	SNU-C5	0.807	0.791	318	U-87-MG	0.752	0.749
291	SU-DHL-10	0.712	0.699	319	U251	0.787	0.789
292	SU-DHL-5	0.771	0.769	320	UACC-893	0.742	0.747
293	SU-DHL-8	0.764	0.759	321	UWB1.289	0.757	0.758
294	SU8686	0.774	0.783	322	VMRC-LCD	0.756	0.756
295	SUIT-2	0.76	0.766	323	YH-13	0.778	0.781
296	SW1088	0.699	0.713				
